# Ecohealth Approach to Urban Waste Management: Exposure to Environmental Pollutants and Health Risks in Yamoussoukro, Côte d’Ivoire

**DOI:** 10.3390/ijerph111010292

**Published:** 2014-10-02

**Authors:** Parfait K. Kouamé, Kouassi Dongo, Hung Nguyen-Viet, Christian Zurbrügg, Christoph Lüthi, Jan Hattendorf, Jürg Utzinger, Jean Biémi, Bassirou Bonfoh

**Affiliations:** 1 Unité de Formation et de Recherche des Sciences de la Terre et des Ressources Minières, Université Félix Houphouët-Boigny, 22 BP 582 Abidjan 22, Côte d’Ivoire; E-Mails: parfait.kouame@csrs.ci (P.K.K.); k_dongo@yahoo.fr (K.D.); jbiemi@yahoo.fr (J.B.); 2 Centre Suisse de Recherches Scientifiques en Côte d’Ivoire, Abidjan, Côte d’Ivoire; CSRS, 01 BP 1303 Abidjan 01, Côte d’Ivoire; E-Mail: bassirou.bonfoh@csrs.ci; 3 International Livestock Research Institute, 17A Nguyen Khang Street, Cau Giay, Hanoi, Vietnam; 4 Centre for Public Health and Ecosystem Research, Hanoi School of Public Health, 138 Giang Vo Street, Hanoi, Vietnam; 5 Department of Epidemiology and Public Health, Swiss Tropical and Public Health Institute, P.O. Box 4002 Basel, Switzerland; 6 Department of Epidemiology and Public Health, University of Basel, P.O. Box number, CH-4003 Basel, Switzerland; E-Mails: jan.hattendorf@unibas.ch (J.H.); juerg.utzinger@unibas.ch (J.U.); 7 Sandec-Department of Water and Sanitation in Developing Countries, Swiss Federal Institute of Aquatic Science and Technology, Überlandstrasse 133, CH-8600, Switzerland; E-Mails: christian.zurbruegg@eawag.ch (C.Z.); christoph.luethi@eawag.ch (C.L.)

**Keywords:** Ecohealth, risk factor, waste management, wastewater, tropical diseases, Côte d’Ivoire

## Abstract

Poor waste management is a key driver of ill-health in urban settlements of developing countries. The current study aimed at assessing environmental and human health risks related to urban waste management in Yamoussoukro, the political capital of Côte d’Ivoire. We undertook trans-disciplinary research within an Ecohealth approach, comprised of a participatory workshop with stakeholders and mapping of exposure patterns. A total of 492 randomly selected households participated in a cross-sectional survey. Waste deposit sites were characterised and 108 wastewater samples were subjected to laboratory examinations. The physico-chemical parameters of the surface water (temperature, pH, conductivity, potential oxidise reduction, BOD_5_, COD, dissolved oxygen, nitrates, ammonia and total Kendal nitrogen) did not comply with World Health Organization standards of surface water quality. Questionnaire results showed that malaria was the most commonly reported disease. Diarrhoea and malaria were associated with poor sanitation. Households having dry latrines had a higher risk of diarrhoea (odds ratio (OR) = 1.8, 95% confidence interval (CI) 1.2–2.7) compared to latrines with septic tanks and also a higher risk for malaria (OR = 1.9, 95% (CI) 1.1–3.3). Our research showed that combining health and environmental assessments enables a deeper understanding of environmental threats and disease burdens linked to poor waste management. Further study should investigate the sanitation strategy aspects that could reduce the environmental and health risks in the study area.

## 1. Introduction

Sanitation deficiency causes environmental and health threats in developing countries. Managing sanitation properly contributes to reducing mortality from diarrhoeal diseases by 65% and morbidity by 26% [1]. About 2.2 million people in developing countries die yearly from diseases associated with lack of safe drinking water, lack of adequate sanitation and poor hygiene [1]. Sanitation management and infrastructure selection must cope with economic development and population growth [2]. A recent study showed that the replication of centralized water, energy and cost-intensive technologies has proved ineffective in resolving the complex water-related problems resulting from rapid urbanization in developing countries [3]. In Somalia, Bella and Vaccari stated that the solutions proposed for waste management are often a mere copy of technical devices adopted in developed countries and that they are not selected on the basis of the actual context of where they are going to be used [4]. Waste management has different components, such as municipal authorities, local government, populations, and communication plans [5,6].

Trans-disciplinary approaches have been developed to improve waste management sector and investigate services’ quality [7]. For example, social network analysis and stakeholder analysis methods were applied to understand actors' role and actions, and to analyse driving forces and existing coordination among stakeholders. Additionally, the influence of policy and evidence generation for technical stakeholders were analysed, by integrating ecosystem, social, and health dimensions [8,9]. Although these provided certain insights, there remain shortcoming in the understanding of disease exposures due to waste management and their determinants factors, such as waste and wastewater discharge sites. Especially, in sub-Saharan Africa, the lack of sanitation is critical in countries which must implement intervention measures under severe resource constraints. Developing adaptable and robust strategies for assessing the overall performance of the waste management system and its impacts are necessary.

In urban settings of Côte d’Ivoire, wastes are illegally dumped, as described elsewhere [10]. Untreated wastewaters were discharged into the watershed in the economic capital Abidjan and the political and administrative capital Yamoussoukro. The inadequacy of urban sanitation technologies for waste management causes environmental and human health consequences [1,11]. Water for irrigating that is of poor quality might cause serious health risks, particularly diarrhoea and typhoid fever [12]. In the study area, wastewaters from drainage network and lakes were used for irrigating lettuce and vegetables. Urban farming activities increase the health risks because of the poor water quality used for irrigation. It is widely acknowledged that water and sanitation measures remain critically important for people’s health in developing countries. Cairncross and colleagues [13] showed that around 4.2% of all deaths could be prevented annually if everyone practised appropriate hygiene, and had access to reliable sanitation and drinking water. The control strategies were based on the water quality improvement and availability, the hygiene promotion, the reduction of contact with infected water and access to sanitation [14,15,16,17]. This study aimed at assessing the disease burdens related to unsustainable urban waste management in order to provide the information necessary to develop appropriate measures and practical interventions that could improve the population’s well-being in Yamoussoukro. A trans-disciplinary approach was undertaken to improve health and sanitation conditions.

## 2. Material and Methods

### 2.1. Study Area

**Figure 1:**
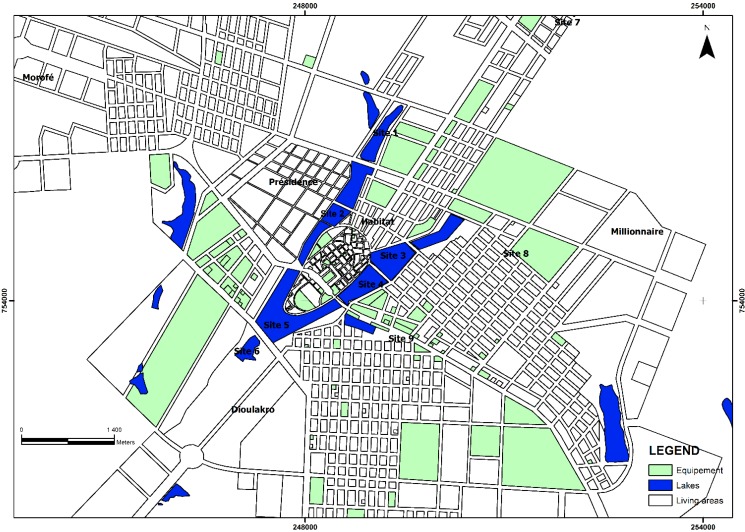
The study area, the city of Yamoussoukro in Côte d’Ivoire.

The current study was carried out in Yamoussoukro, located about 240 km of Abidjan. It is located at 6°47ꞌ–6°52ꞌN latitude and 5°22 ꞌ–5°23ꞌ W longitude and covers approximately 28,000 ha (Figure 1). The population of Yamoussoukro is estimated at 300,000 [18]. The climate is tropical with four seasons. The long dry season occurs from December to May and a shorter dry season spans from July to October. The long rainy season lasts from May to July and the shorter rainy season occurs in October and November. The yearly precipitation varies from 900 mm to 1200 mm and the average humidity is from 80% to 95%. The temperature ranges from 19 °C to 35 °C. The elevation is around 200 m. The main local rivers are the N’Zi and Marahouet and they are connected to the Bandama River. Drinking water sources are taps and wells, and there is generally poor groundwater quality.

About 90% of households use septic tanks. Most of the faecal sludge is removed by private actors, and in some cases manually by traditional scavengers [19]. Solid waste and health management is done by the local authorities. The lake waters, covering 140 ha, currently receive all the wastewater from anthropogenic activities [20]. Urban agriculture uses the contaminated lake waters and people also fish in the lakes. The health department of the city of Yamoussoukro yearly reports many cases of bloody diarrhoea, typhoid fever, and malaria.

### 2.2. Methodological Framework

The methodological framework of our study is an adaptation of the Ecohealth approach. In brief, an Ecohealth approach represents a set of methodological and conceptual steps that enable a better understanding of complex interactions between the various components of a social-ecological system (e.g., physico-chemical factors, inorganic or organic substances, the environment, and the population), and how these interactions influence human health and well-being. It also seeks to identify ecosystem management strategies that contribute to improving the health and living conditions of the human population and the sustainability of the ecosystems in which they live [21]. This approach integrated physical environment, health status, social, cultural, and economic environment aspects [22]. A recent study conducted by Charron developed a frame based on six principals, namely (i) systems thinking; (ii) trans-disciplinary research; (iii) participation, (iv) sustainability; (v) gender and equity; (vi) and knowledge-to-action [23]. Then, it was found that the Ecohealth approach implementation enabled the comprehensive rapid assessment of environmental and health risk exposure pathways and the development of intervention plans.

**Figure 2:**
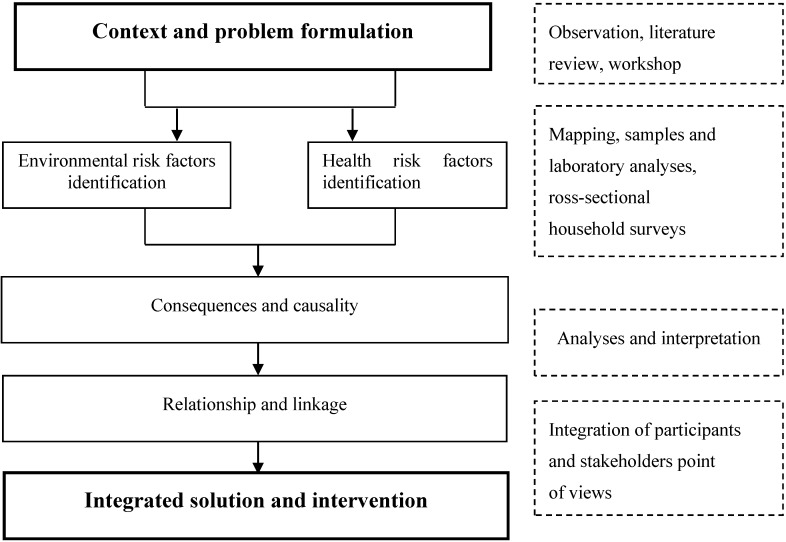
Framework for developing an integrated approach for environmental and health risks factors analysis.

For this study, we applied an Ecohealth approach, which focused only on environmental, health, and opinions assessment, in order to inform stakeholders in the city of Yamoussoukro. We conducted water quality and risk factor assessments. We also used the Ecohealth approach to analyze environmental and health threats. As seen in Figure 2, the components analysed in the Ecohealth approach were: (i) problem description; (ii) mapping of environmental and health risk factors at the city level; (iii) identification of potential causes and consequences; and (iv) setting a specific intervention plan with stakeholders. This allowed for a better description of the risk patterns in the study area and their distribution and mitigation.

The purpose of using social dimensions in this research was to show how access to safe sanitation conditions and the improvement of waste management contribute to enhanced environmental and human health in the area. Social equity is here defined as equal access sanitation and the social determinants of health depend on living in a safe environment. A workshop was held with important stakeholders to receive their opinions about the environment and health conditions in the study area.

Action plans are then elaborated based on stakeholders’ outputs and relevant and specific actors for its implementation have been identified.

### 2.3. Participatory Workshop

A one-day participatory workshop was held in February 2011, and main participants were selected based on the research purpose and relevant implications on waste management in the city of Yamoussoukro. Overall, 35 stakeholders from waste management actors, health, urban planning, and municipal sectors of Yamoussoukro, as well as non-governmental organisations, farming communities, and the general population participated. The main focus of the workshop was to understand the requirements and constraints of providing sanitation and health services in the study area in order to facilitate the implementation of future intervention solutions. The workshop was based on a standard process, comprising five main steps: (i) identification of key participants; (ii) study goal presentation to the participants; (iii) small group discussion; (iv) plenary discussion, where the representative of each group reported main findings; (v) and synthesis of key group findings. In this last step, the facilitator made a final list of recommendations for further actions to be conducted.

### 2.4. Mapping Approach for Investigating Environmental Risk Factors

Mapping of indiscriminate solid waste dumping and wastewater discharge sites in the city shows their spatial distribution and exposures routes. The main critical mapping points of the study were geo-referenced by cross-sectional geographical surveys that were conducted from July 2011 to August 2011, with a GPSMAP 62 (Kansas, USA). The points were located using Universal Transverse Mercator (UTM) coordinates. The investigation focused on solid waste and wastewater flows, the availability of sanitation facilities (latrines), and the location of waste (outside of official sites, in close proximity to houses, and anthropogenic activities, *etc*.).

### 2.5. Cross-Sectional Household Surveys

Cross-sectional surveys were conducted from July 2011 to August 2011 in 492 households. The main hypothesis of the surveys was that disease occurrence in the study area depends on household behaviour and hygiene practices, and waste management infrastructure or the lack thereof. The sample size was calculated with a precision of 5% and a 95% confidence level, based on Equation (1) [24].

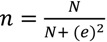
(1)


In this equation, *n* represents the sample size and *N* the number of inhabitants in the study area (300,000) and e represents the acceptable sampling error (5%). With Google Maps, households were selected randomly, so that they covered the entire study area. All household members above 14 years of age were eligible. The household questionnaires were addressed preferably to the women in the household, because they normally manage the handling of solid waste and wastewater. Questionnaires at the household and personal level were administered to all household members. Surveys collected general demographic information, as well as information on socioeconomic status, households’ general sanitary conditions, access to water and sanitation, and satisfaction with waste management services. Table 1 presents the main variables analysed in the surveys. Health status was assessed by asking if any household member suffered from malaria, typhoid fever, or diarrhoea during the past 2 years.

### 2.6. Sampling Methods and Laboratory Analyses

#### 2.6.1. Wastewater Sampling Method

Samples were taken from nine lake water and anthropogenic wastewater sites. The sites were chosen according to key activities (e.g., urban farming, open-space restaurants, hospitals, *etc.*) and their geographical location (density area), and whether these, caused the poor water quality in the study area due to the human activities, and poor sanitation practices and facilities. There were six in lake water sites, two in wastewater treatment plants and one in the open drainage network. A total of 12 fortnightly water sampling campaigns were conducted between November 2011 and January 2012 (dry season) and between May 2012 and July 2012 (rainy season). Samples were stored in plastic bottles and placed in an ice-packed cooler according to the “Rodier Guidelines for Sampling and Water Quality in Environment Monitoring” (reference method: FD T90-523-1 of 2008, T90-523-1) [25]. Overall, 108 water samples were collected and transferred to a nearby laboratory for analysis. Temperature (T), pH, conductivity (Cond), oxidation-reduction potential electricity (Redox), and dissolved oxygen (DO) were measured *in situ*, using a portable multiple parameter WTW 350i/SET (no. 2F40-114B0E; Weilheim, Germany) [26].

**Table 1:** Main variables for understanding disease occurrences related to household exposure.

Variables	Description	Indicator	
		Health	Environment
Socioeconomic status	Poor or wealthy	x	x
Living place	Residential area or poor settlement	x	x
Solid waste discharged	Close/far (10–50 m) to the household and surrounding activities (restaurants)	x	x
Wastewater released	In open drainage or septic tanks	x	x
Household drinking water source	Wells or taps	x	x
Access to safe sanitation facilities	Pit latrine or septic tank influence disease occurrences?	x	
Type of reference diseases	Malaria, typhoid, diarrheal cases	x	
Vegetables crops from the urban agriculture using contaminated wastewater	Salad crop, carotene, cucumber, tomatoes	x	

#### 2.6.2. Physico-Chemical Analyses

Chemical analyses were conducted to determine environmental pollution and the water quality. The targeted parameters focused on anthropogenic contaminants, especially nitrogen components (NO_3_^-^, NH_4_^+^, and TNK). Indeed, nitrogen is an essential nutrient for aquatic organisms, and plants absorb it in the form of nitrates. These constitute an essential part of the inorganic nitrogen content in water. The nitrogen concentrations (e.g., ammonium nitrogen) of around 1.5–2 mg N/L in surface water are mostly linked to anthropogenic activities and leaching from agricultural land [2,27]. Lake water and wastewater samples were analysed to determine the physico-chemical parameters BOD_5_ and COD. The BOD_5_ parameter was analysed using a BOD Track (HACH version 26197-94; Loveland, CO, USA) [28], and TNK and COD parameters were measured by the titrimetric method. The nutrients NO_3_^-^ and NH_4_^+^ were measured using a spectrophotometer (HACH DR2400; Loveland, CO, USA) [29].

Table 2 summarises the analytical methods used to determine the physico-chemical parameters.

### 2.7. Statistical Analysis

Household survey data were recorded in EpiInfo version 3.5.1 (Centers for Disease Control and Prevention; Atlanta, GA, USA). The data were analysed using R software version 3.0.1 (R Development Core Team; Vienna, Austria) to assess the link between water pollution patterns and human activities. Logistic regression was used to explore the links between environmental determinants and exposures and targeted diseases, and to examine the factors that influence disease dynamics (e.g., access to sanitation). Odds ratios (ORs) were calculated to approximate relative risk and are presented with 95% confidence intervals (CIs). Statistical models were developed to assess the effect of the socioeconomic status (SES) of the population, the type of living place (TLP), the availability of waste facilities (WF), and sanitation services accessibility (SSA) had on the dynamic of the occurrence of diseases, such as diarrhoea, malaria, and typhoid fever. Additionally, a descriptive statistical analysis was done to determine the physico-chemical parameters (mean and standard deviation).

**Table 2:** Water quality parameters and analytical methods.

Parameters	Abbreviation	Unit	Analytical Techniques
Temperature	Temp	°C	Multiple parameter pocket, WTW 350i
Electrical conductivity	Cond	μS/cm	Multiple parameter pocket, WTW 350i
pH	pH	-	Multiple parameter pocket, WTW 350i
BOD	BOD_5_	mg/L	5-day incubation at 20 °C with BOD Track, HACH version 26197-94
Chemical oxygen demand	COD	mg/L	Titrimetric method with dichromate potassium NF T 90-101-2001
TNK	TNK	mg/L	TNK method (Norm ISO 5663-1984) after mineralisation with selenium
Dissolved oxygen	DO	mg/L	Multiple parameter pocket, WTW 350i
Ammonium nitrogen	NH_4_^+^	mg/L	Salicylate Method, HACH DR/2400 programme 385N
Nitrate	NO_3_^−^	mg/L	Reduction Method with cadmium, HACH DR/2400 program 353N

## 3. Results

### 3.1. Perceived Environmental and Health Threats by Population and Spatial Distribution of Waste

Results of the participatory workshop revealed that most of the participants feel threatened by the degradation of environmental and health conditions in the study area. Moreover, they stressed the negative effects of poor management of wastewaters and solid wastes on lake water and groundwater contamination and on the dynamics of disease burdens, such as diarrhoea and typhoid fever. However, the consideration of the health risks associated with vegetables consumption is not well understood in the study area. During the workshop, a representative of the farmers said: “*I eat salad vegetable harvested using wastewater and lake water, and I have never been sick*”. As recommendations, participants suggested the construction of sanitation infrastructures to properly treat the urban wastewater and collect solid waste regularly and equitably in the study area.

**Figure 3:**
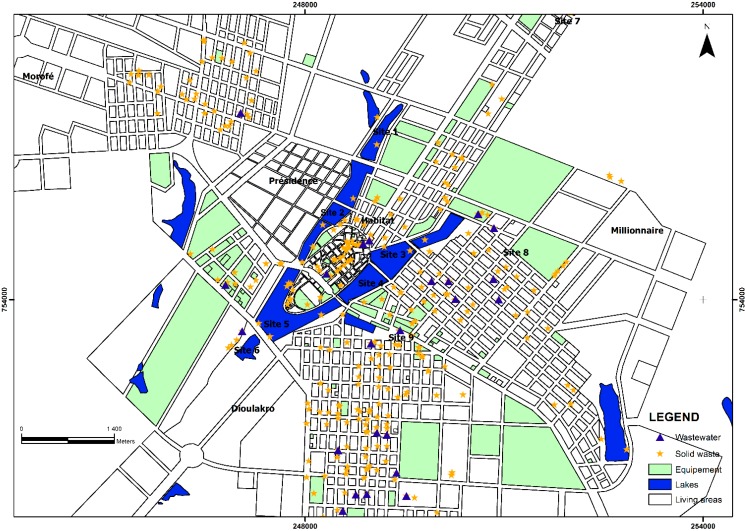
Mapping of disease burdens pathways, and the lack of solid waste and wastewater management in Yamoussoukro.

Additionally, the mapping of indiscriminate solid waste deposit sites and untreated wastewater discharge points enabled the identification of the main exposed settlements and facilitated the subsequent intervention. In addition, the results of field observations, coupled with the geographical cross-sectional surveys, revealed various exposure routes in the study area. Wastewater, solid wastes openly dumped in close proximity to houses, markets, and restaurants were observed. These lead to an increase in exposure to disease. However, there was a disparity in access to the sanitation facilities in the settlements, where many wastewater discharge sites were observed. Figure 3 shows the poor management of both solid waste and wastewater in the study area.

### 3.2. Characteristics of the Study Population

The households surveyed (n = 492) were composed of 74.2% females and 25.8% males. Our investigation showed that the education level of the persons interviewed was estimated at 13.7% who had higher education, 34.5% had secondary schooling, 18.4% had primary schooling, and 32.8% had no schooling. A total of 2,786 people were living in the households and 602 were aged <5, 505 were 6–10, 452 were 11–15, and 1732 were >15 years of age. The results showed that the consumption of fresh vegetables once a month, namely of salad, cucumbers, and tomatoes, by the surveyed households was estimated at 75.6% for salad, 85.8% for cucumber, and 95.5% for tomatoes during the study period.

### 3.3. Environmental Pollution

Table 3 and Table 4 present the physico-chemical characteristics of the water samples obtained from the nine sample sites. We found considerable spatial and temporal heterogeneity. The pH values of the samples ranged from 6.6 to 7.7. The highest pH value was found in samples from sample site 2 (6.4–9.0). In this study, the results of the Dissolved Oxygen (DO) varied from 0.2 mg/L to 6.5 mg/L, and were low in our investigated wastewater samples. The lowest value of DO was obtained in t sample site 9 (1.4 mg/L). The nitrogen components, mainly nitrates and ammonia concentrations, varied from 0.6 mg/L to 8.3 mg/L for NO_3_^−^ and from 0.1 mg/L to 33 mg/L for NH_4_^+^, respectively. The nitrates concentration is higher in sample site 1 and sample site 9, and ammonia is high at the wastewater treatment plant (WTP), sample site 7. The COD varies from 44 mg/L to118 mg/L and COD values from the WTP of sample site 8 and sample site 7 and from the lake water of sample site 2 were high, especially, during the dry season. The BOD_5_ varied from 20 mg/L to 42 mg/L and was relatively high at the WTP of sample site 8 and sample site 7, in the lake water of sample site 2 and the drainage water of sample site 9, and was estimated at 24.6 mg/L to 39.3 mg/L, respectively, during the dry season. The results of dissolved minerals at each station are expressed by the conductivity, and the values varied from 200 μS/cm to 436 μS/cm. The redox oxidise potential varied from 43 mV to 63 mV, and the higher values were observed during the dry season.

**Table 3:** Physico-chemical parameters characteristic in dry season (mean, standard deviation).

Sample Site	Location	pH	DO (mg/L)	Cond (μS/cm)	NO_3_^−^ (mg/L)	NH_4_^+^ (mg/L)	BOD_5_ (mg/L)	COD (mg/L)	TNK (mg/L)
1	Guiglo	6.9 ± 1.2	2.2 ± 2.8	291.7 ± 98.2	8.3 ± 9.6	0.3 ± 0.2	17.5 ± 5.1	44.8 ± 14.4	1.5 ± 0.2
2	Presidence	7.7 ± 1.2	4.7 ± 2.4	211.6 ± 23.8	1.0 ± 0.6	0.1 ± 0.1	24.6 ± 5.9	58.1 ± 13.3	1.3 ± 0.1
3	Ngokro	7.0 ± 0.1	1.7 ± 1.8	326.2 ± 56.4	1.0 ± 0.2	0.1 ± 0.1	27.3 ± 12.9	58.6 ± 19.8	1.3 ± 0.2
4	Cyclone	7.3 ± 0.2	3.0 ± 1.1	290.0 ± .9	2.5 ± 1.0	0.1 ± 0.1	23.1 ± 8.0	53.3 ± 18.7	1.4 ± 0.2
5	CHR	7.6 ± 0.6	3.5 ± 1.7	298.7 ± 29.8	0.7 ± 0.5	0.2 ± 0.1	18.8 ± 5.0	69.9 ± 21.3	7.5 ± 1.9
6	Basilique	7.1 ± 0.2	2.5 ± 1.4	436.0 ± 13.8	1.5 ± 0.7	0.6 ± 0.8	19.6 ± 7.9	78.0 ± 15.7	1.3 ± 0.5
7	INP-HB	7.3 ± 0.2	2.6 ± 1.5	382.8 ± 53.6	1.8 ± 1.1	23.1 ± 5.6	24.1 ± 12.6	70.3 ± 29.8	2.7 ± 0.7
8	Lycée Scient.	7.0 ± 0.1	0.2 ± 0.1	436.0 ± 13.8	2.2 ± 1.1	10.7 ± 2.0	25.0 ± 11.3	75.1 ± 25.6	1.9 ± 0.6
9	CIE	6.9 ± 0.1	1.4 ± 2.1	396.8 ± 151.5	8.2 ± 5.5	6.7 ± 6.8	39.3 ± 5.8	65.3 ± 19.8	1.5 ± 0.1

**Table 4:** Physico-chemical parameters characteristic in rainy season (mean, standard deviation).

Sample Site	Location	pH	DO (mg/L)	Cond (μS/cm)	NO_3_^−^ (mg/L)	NH_4_^+^ (mg/L)	BOD_5_ (mg/L)	COD (mg/L)	TNK (mg/L)
1	Guiglo	6.8 ± 0.2	1.8 ± 0.3	252.3 ±27.9	1.0 ± 1.6	0.1 ± 0.1	18.2 ± 2.3	43.7 ± 4.3	1.5 ± 0.3
2	Presidence	7.7 ± 1.0	4.1 ± 1.5	247.2 ± 20.6	0.8 ± 1.4	0.1 ± 0.1	30.6 ± 3.9	74.0 ± 18.1	1.6 ± 0.2
3	Ngokro	6.6 ± 0.1	3.0 ± 2.4	258.3 ± 46.0	7.9 ± 8.5	1.2 ± 2.1	26.5 ± 4.3	43.6 ± 6.9	1.3 ± 0.2
4	Cyclone	7.7 ± 0.6	3.6 ± 1.0	342.2 ± 35.2	0.6 ± 0.6	0.1 ± 0.0	31.4 ± 6.3	58.7 ± 16.8	1.7 ± 0.2
5	CHR	7.5 ± 0.4	3.1 ± 2.1	325.2 ±15.1	0.7 ± 0.4	0.3 ± 0.2	20.4 ± 4.1	51.9 ± 16.0	1.5 ± 0.3
6	Basilique	6.9 ± 0.2	1.5 ± 1.0	362.5 ± 41.7	1.1 ± 0.8	8.0 ± 6.6	22.9 ± 3.4	42.4 ± 8.4	1.2 ± 0.4
7	INP-HB	7.5 ± 0.5	6.5 ± 10.4	291.5 ± 151.3	2.6 ± 2.2	33.4 ± 17.2	39.0 ± 13.9	85.4 ± 18.5	3.0 ± 1.5
8	Lycée Scient.	7.0 ± 0.1	4.8 ± 11.0	433.3 ± 26.5	1.7 ± 2.1	13.5 ± 8.5	41.9 ± 14.7	118.0 ± 30.2	1.9 ± 0.5
9	CIE	6.9 ± 0.1	1.4 ± 2.1	396.8 ± 151.5	8.2 ± 5.5	6.7 ± 1.8	21.3 ± 5.8	77.1 ± 35.8	1.5 ± 0.1

### 3.4. Prevalence of Malaria and Water-Borne Diseases

**Figure 4:**
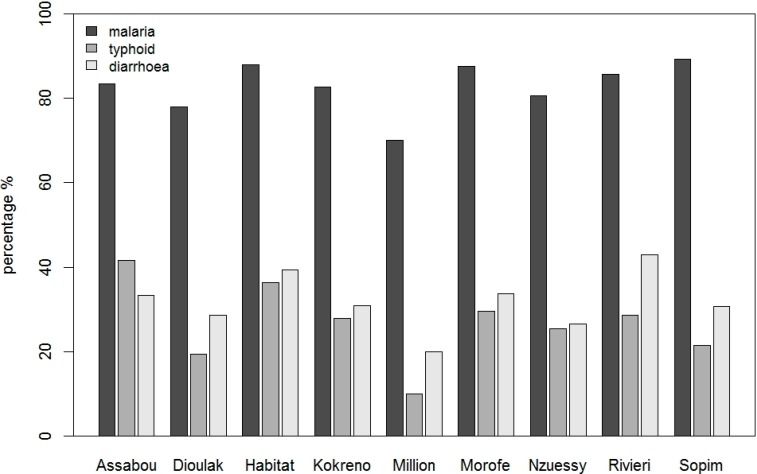
Proportion of households reporting disease occurrences in the past 2 years in settlements of Yamoussoukro.

More than 80% of the households reported that at least one household member suffered from malaria during the past 2 years. There was some variation among the settlements (ranging from 70% to 89%) but malaria was frequently reported in all areas (Figure 4). In addition about one fourth of the households reported episodes of typhoid fever. The proportion was highest in Assabou and Habitat with a proportion of 40% and 36% respectively. The proportion of households reporting diarrhoea was surprisingly low (31%), which might be related to recall bias since the recall time was quite long for remembering past diarrhoea episodes. Households in Millionaire reported the lowest proportion of all three diseases.

### 3.5. Disease Risk Factors

The results obtained from the cross-sectional household surveys described the main drinking water sources in the study area. Water types have an impact on the water-borne disease dynamics. The main water supply source is tap water, at 80%. The accessibility to tap water, resale water, and traditional well water is estimated to be 70%, 18%, and 41%, respectively. Alternative sources of water (wells and resale) were used mostly by poor households for domestic activities, such as bathing and washing clothes. Table 5, Table 6 and Table 7 summarize the results of exposure factors linked to disease dynamics.

**Table 5:** Typhoid dynamics, risk factors related to urban waste management in Yamoussoukro.

Characteristic ^1^	Healthy ^2^	Diseased ^3^	OR	95% CI	*p*-value
**Housing Condition (n: 435)**					
Poor/common	57% (184)	68% (78)	Ref.		
economic/residential	43% (137)	32% (36)	0.6	0.4–1.0	0.04
**Income Per Month (US$ n: 462)**					
Above 200	40% (58)	42% (22)	Ref.		
100 to 200	45% (65)	34% (18)	0.7	0.4–1.5	0.39
Below 100	16% (23)	25% (13)	1.5	0.6–3.4	0.35
**Drinking Water Source (n: 487)**					
Tap	68% (232)	73% (88)	Ref.		
Well	29% (99)	26% (31)	0.8	0.5–1.3	0.43
Excl. resale	3% (10)	2% (2)	0.5	0.1–2.1	0.41
**Sanitary Access (n: 467)**					
Septic tank	59% (203)	51% (62)	Ref.		
Dry latrine	38% (131)	44% (54)	1.3	0.9–2.1	0.17
None	3% (11)	5% (6)	1.8	0.6–4.9	0.27
**Waste Water Disposal (n: 467)**					
Street	33% (113)	41% (50)	ref		
Septic tank	10% (35)	9% (11)	0.7	0.3–1.5	0.37
Open drainage	18% (61)	11% (13)	0.5	0.2–0.9	0.04
Other	39% (136)	39% (48)	0.8	0.5–1.3	0.34
**Solid Waste Disposal (n: 434)**					
Inside home	74% (240)	70% (78)	Ref.		
Outside home	26% (83)	30% (33)	1.2	0.8–2.0	0.41

Notes: **^1^** **Characteristic,** described the risk factor assessed in the study; **^2^** **Healthy**, the percentage of the healthy people in surveyed households; **^3^** **Disease,** the percentage of the sick people in the surveyed households. Ref. indicates reference.

**Table 6:** Malaria dynamics, risk factors related to urban waste management in Yamoussoukro.

Characteristic	Healthy	Diseased	OR	95% CI	*p*-value
**Housing Condition (n: 438)**					
poor/common	61% (43)	60% (221)	Ref.		
economic/residential	39% (27)	40% (147)	1.1	0.6–1.8	0.83
**Income Per Month (US$ n: 200)**					
above 200	41% (13)	40% (67)	Ref.		
100 to 200	47% (15)	40% (68)	0.9	0.4–2.0	0.76
below 100	12% (4)	20% (33)	1.6	0.5–6.0	0.44
**Income Per Month (US$ n: 465)**					
Tap	66% (50)	70% (272)	Ref.		
Well	30% (23)	28% (108)	0.9	0.5–1.5	0.59
excl. resale	4% (3)	2% (9)	0.6	0.2–2.6	0.38
**Sanitary Access (n: 470)**					
Septic tank	69% (53)	54% (213)	Ref.		
Dry latrine	29% (22)	42% (165)	1.9	1.1–3.3	0.02
None	3% (2)	4% (15)	1.9	0.5–12.1	0.42
**Waste Water Disposal (n: 470)**					
Street	34% (26)	35% (138)	Ref.		
Septic tank	10% (8)	10% (38)	0.9	0.4–2.3	0.8
Open drainage	17% (13)	16% (62)	0.9	0.4–1.9	0.77
Other	39% (30)	39% (155)	1	0.6–1.7	0.93
**Solid Waste Disposal (n: 437)**					
Inside home	66% (49)	75% (271)	Ref.		
Outside home	34% (25)	25% (92)	0.7	0.4–1.2	0.14

**Table 7:** Diarrhoea dynamics, risk factors related to urban waste management in Yamoussoukro.

Characteristic	Healthy	Diseased	OR	95% CI	*p*-value
**Housing condition (n: 435)**					
poor/common	58% (176)	65% (86)	Ref.		
economic/residential	42% (127)	35% (46)	0.7	0.5–1.1	0.17
**Income per month (US$ n: 462)**					
above 200	40% (52)	41% (28)	Ref.		
100 to 200	45% (59)	35% (24)	0.8	0.4–1.5	0.41
below 100	15% (19)	25% (17)	1.7	0.7–3.7	0.21
**Drinking water source (n: 487)**					
Tap	68% (219)	71% (101)	Ref.		
Well	28% (91)	27% (39)	0.9	0.6–1.4	0.75
excl. resale	3% (10)	1% (2)	0.4	0.1–1.7	0.29
**Sanitary access (n: 467)**					
Septic tank	61% (197)	47% (68)	Ref.		
Dry latrine	35% (114)	49% (71)	1.8	1.2–2.7	0.004
None	4% (12)	3% (5)	1.2	0.4–3.4	0.73
**Waste water disposal (n: 467)**					
Street	32% (102)	42% (61)	Ref.		
Septic tank	10% (33)	9% (13)	0.7	0.3–1.3	0.25
Open drainage	17% (54)	14% (20)	0.6	0.3–1.1	0.12
Other	41% (134)	35% (50)	0.6	0.4–1.0	0.04
**Solid waste disposal (n: 434)**					
Inside home	73% (222)	74% (96)	Ref.		
Outside home	27% (83)	26% (33)	0.9	0.6–1.5	0.73

Dry latrines were associated with a higher risk for diarrhea compared to latrines with a septic tank (OR = 1.8, 95% CI 1.2–2.7) and also a higher risk for malaria (OR = 1.9, 95% CI 1.1–3.3). Low SES in terms of monthly income was associated with a higher risk to all three diseases with ORs between 1.5 and 1.7. However, the association was not statistically significant since many interviewees refused to answer questions concerning household incomes. Therefore only few households were in this category. Lower risk of typhoid fever was identified for people living in better residences (OR = 0.6, 95% CI 0.4–1.0) and for people discharging their wastewater in the open drainage system and not in the street (OR = 0.5, 95% CI: 0.2–0.9). Wastewater disposal in the streets was also associated with a higher risk of diarrhea compared to other methods of disposal (OR = 0.6, 95% CI 0.4–1.0).

## 4. Discussion

Applying a trans-disciplinary research approach in developing countries could help to improve their health and environmental conditions. The economic development in these countries is associated with poor waste management and agricultural practices, which contribute to the degradation of water quality [30]. For this research, a framework to assess human health and environmental aspects was developed, by integrating the study of urban waste management systems, environmental sanitation policies, and anthropogenic practices. Especially, in the context of Côte d’Ivoire, it was shown that the poor waste management sector was negatively affecting environmental quality and human health and well-being, particularly in urban and peri-urban areas [10]. Thus, there is a pressing need to mitigate the health hazards related to urban waste in these areas, especially in developing countries.

The workshop conducted highlighted the poor waste management. Farmers were not well informed about the health risks linked to the use of wastewater for farming activities. The participants were not satisfied with the current waste management conditions, because of the impacts on environmental degradation and health exposures that were observed. It is recommended that sanitation infrastructure be built and solid waste collection increased in Yamoussoukro. Based on these recommendations of the beneficiaries, an intervention project has been implemented and financially supported by the Volkswagen Foundation. This project allowed to train the population on waste separation at household level and waste reuse and recycling. Organic wastes are currently being composted and used as fertilizer.

A study done by N’Guessan and colleagues discussed the lack of sanitation in the city of Yamoussoukro, and focused on the lake waters quality degradation related to the waste management [20]. Yearly reports by the local health authorities documented cases of malaria, bloody diarrhoea and typhoid fever. The Health Department, for instance, in 2012, reported that there were 30,247 cases of malaria, 3444 cases of diarrhoea, and 206 cases of typhoid fever. Of note, malaria and typhoid fever co-exist in tropical settings, and hence, it is difficult, to clearly differentiate between the two without in-depth clinical or microbiological investigations [17]. Diarrhoeal incidences linked to water, sanitation, and hygiene have been well documented in many literature reviews and in the databases of international agencies [14,15,31,32]. The current research addressed the potential health effects and environmental degradation due to solid waste and untreated wastewater being openly dumped and released into the environment, and linked the occurrences of water-borne diseases and the contamination of the lake waters in the city of Yamoussoukro. The participatory workshop revealed that the local population is concerned about environmental threats and ill-health that might be caused by poor water quality in the study area. The main factors determining poor urban waste management, exposure routes and environmental degradation have been assessed. The mapping of uncontrolled solid waste deposit and wastewater release sites in the city of Yamoussoukro revealed the potential health effects due to the lack of solid waste removal, and the difficulty of accessing sanitation services and the lack of equity regarding such services in some settlements. The mapping highlighted the spatial distribution of critical points, such as waste disposal sites and wastewater release points. Especially in Habitat, Dioulakro, and Morofé neighbourhoods of Yamoussoukro, many critical control points (CCP) related to solid wastes were observed. It is conceivable that the deficiency of sanitation strategies and poor management of solid waste and wastewater are linked to poor health. Indeed, in Indonesia, for example, it was shown how safe water and sanitation management helped to reduce water-borne diseases [1].

Indiscriminate waste disposal in the study area affected the population’s health condition. People living in the study area were suffering from malaria, typhoid, and diarrhoea. The results of household cross-sectional surveys enabled the research team to assess the level of malaria, typhoid, and diarrhoea caused by the emission of hazards (microorganisms and contaminants from urban waste and wastewater). Some disparities in the disease dynamics in the study area were highlighted. Households having on-site sanitation with dry latrines were most exposed to diarrhoea and malaria than those using latrines with septic tanks. Statistical analyses showed that income influenced the process of getting diseases. A low risk of typhoid was observed for people living in better residential areas. The association was not statistically significant, however, since many interviewees refused to answer the question about their income.

Then, results of diseases occurrences were obtained from surveyed households and were based on clinical analyses obtained after diagnostic from hospital. Information concerning the relation between waste disposal, diseases, and income has to be considered in planning processes, in order to reduce the health threats related to waste management. This study has shown that the high levels of malaria and typhoid were linked to the poor management of waste collection and to wastewater collection deficiencies, reflecting the lack of sanitation infrastructure in the study area. Therefore, the interaction among social aspects (lack of infrastructures), environmental (poor urban waste management), and health status (typhoid, malaria) is the key determinant of population well-being in the study area.

In Salvador, Brazil, a study found that establishing decentralized sanitation technology contributes to declining the prevalence of diarrhoea by up to 40% [14]. Results of our logistic regression analysis showed that malaria and diarrhoeal diseases were most influenced by the poor management of sanitation conditions. Households in poor neighbourhoods with septic tanks and dry latrines contributed to this, because of illegal connections with open drainage. In the current study, diarrhoea was attributable to lack of sanitation and to the poor quality of wastewater used for irrigation and accidental contact with indiscriminate wastes and wastewaters openly dumped in the study area. Interestingly, 80% of the population in Yamoussoukro have access to tap water and 41% to wells. Our research showed that the water supply origins at the household level did not affect the occurrence of diarrhoea. Many studies have found that diarrhoeal infections are caused by the use of wastewater or by drinking contaminated water and/or consumption of contaminated food [33]. Pathogens, such as *Escherichia coli*, could be involved in diarrhoeal contamination due to runoff and the use of wastewater for irrigation, as well as because of the accidental release of effluents in the open drainage network by poorly managed sanitation facilities [14,34].

Understanding the physico-chemical parameters of water quality assessments is necessary for developing sustainable waste management strategies and adaptable intervention measures. Parameters, such as pH, NO_3_^−^, NH_4_^+^, and TNK, BOD_5_, and COD were measured to monitor the quality of water in a given study area [30]. pH affects chemical and biological processes and temperature affects the availability of oxygen concentration in the water [35]. Results of the laboratory analyses showed that the pH values of drainage water and lake waters ranged between 6.7 and 6.9 and from 6.8 to 7.7, respectively. According to Kowalkowski, the pH values of surface water should be in the acceptable limit of 6.5–8.5 [36]. However, the pH of Lake Ngokro was 6.2; above the standard limit of water quality [36]. The degradation of organic matter in the water consumes the available DO and results in high values of BOD_5, _NH_4_^+^, and COD. BOD is measured to look at the quantity of oxygen consumed by microorganisms during the decomposition of organic matter. The DO concentration of the lake water quality in Yamoussoukro is above the thresholds put forth by the World Health Organization (WHO) (limit of 4–10 mg/L) [35]. The WHO BOD_5_ value for unpolluted natural water is to be below 5 mg/L. The results of BOD_5_ obtained for this study were above 17 mg/L, and described the water pollution in this study area. Our results coincided with those in Jakarta in terms of the poor quality of water. Statistical analyses showed that the problems of accessibility and equity of access to safe sanitation facilities and solid waste removal rates considerably affected disease occurrences in the study area. The nitrogen trends were attributable to flows of organic matter from anthropogenic activities and the use of fertilizers for agricultural practices close to lake waters [37].

## 5. Conclusions

Our study targeted the need to develop and implement a practical tool to enhance people’s health and environmental sanitation conditions by assessing potential threats in the study area. These results could be a first step toward the development of a network that would share information among stakeholders, policy makers, and the local population in order to improve people’s well-being in the city of Yamoussoukro. This approach is flexible and could be used for a rapid assessment of environmental and health conditions in developing countries. Biases linked to the lack of information on illiteracy constituted a limitation in this study as well as impacts of knowledge-to-action. There is a need to further analyse sanitation infrastructure implementation aspects, by assessing the technological feasibility, financial reliability, and sustainable viability and to evaluate the impacts of interventions.
